# Wound Healing Potential of Spirulina Protein on CCD-986sk Cells

**DOI:** 10.3390/md17020130

**Published:** 2019-02-22

**Authors:** Ping Liu, Jeong-Wook Choi, Min-Kyeong Lee, Youn-Hee Choi, Taek-Jeong Nam

**Affiliations:** 1 Department of Food Science and Nutrition, Pukyong National University, Busan 48513, Korea; liuping198909@163.com; 2 Institute of Fisheries Sciences, Pukyong National University, Busan 46041, Korea; wook8309@naver.com (J.-W.C.); 3633234@hanmail.net (M.-K.L.); unichoi@pknu.ac.kr (Y.-H.C.); 3 Department of Marine Bio-Materials and Aquaculture, Pukyong National University, Busan 48513, Korea

**Keywords:** spirulina crude protein, dermal fibroblasts, wound healing, cell cycle, PI3K/Akt signaling pathway

## Abstract

Wound healing is a dynamic and complex process. The proliferation and migration of dermal fibroblasts are crucial for wound healing. Recent studies have indicated that the extracts from *Spirulina platensis* have a positive potential for wound healing. However, its underlying mechanism is not fully understood. Our previous study showed that spirulina crude protein (SPCP) promoted the viability of human dermal fibroblast cell line (CCD-986sk cells). In this study, we further investigated the wound healing effect and corresponding mechanisms of SPCP on CCD-986sk cells. Bromodeoxyuridine (BrdU) assay showed that SPCP promoted the proliferation of CCD-986sk cells. The wound healing assay showed that SPCP promoted the migration of CCD-986sk cells. Furthermore, cell cycle analysis demonstrated that SPCP promoted CCD-986sk cells to enter S and G_2_/M phases from G_0_/G_1_ phase. Western blot results showed that SPCP significantly upregulated the expression of cyclin D1, cyclin E, cyclin-dependent kinase 2 (Cdk2), cyclin-dependent kinase 4 (Cdk4), and cyclin-dependent kinase 6 (Cdk6), as well as inhibited the expression of CDK inhibitors p21 and p27 in CCD-986sk cells. In the meanwhile, SPCP promoted the phosphorylation and activation of phosphoinositide 3-kinase (PI3K) and protein kinase B (Akt). However, the phosphorylation of Akt was significantly blocked by PI3K inhibitor (LY294002), which in turn reduced the SPCP-induced proliferation and migration of CCD-986sk cells. Therefore, the results presenting in this study suggested that SPCP can promote the proliferation and migration of CCD-986sk cells; the PI3K/Akt signaling pathway play a positive and important role in these processes.

## 1. Introduction

Skin mainly consists of epidermis and dermis, which acts as the largest organ of the human body against the outside environment [1]. Accordingly, skin wounds disrupt the barrier between the inner body and outer surroundings, and finally lead to severe physical burdens upon human society [2]. Previous studies proved that wound healing is a dynamic and complex process, which requires an interaction between cell migration and cell proliferation relating to the regeneration and replacement of injured tissues [3,4]. Besides, the mechanical support and physical characteristics of the skin are dependent on the dermis [5]. Thus, it is necessary to develop an effective way for promoting the migration and proliferation of dermal fibroblasts.

Microalgae belong to unicellular microorganisms, including any kinds of vitamins, polyunsaturated fatty acids, pigments, polyphenols, polysaccharides, and proteins [6]. Thus, they have many potential health effects, such as antioxidant activity, antiviral activity, and anti-inflammatory activity [7,8]. Among the microalgae, spirulina is rich in excellent source of nutrients [9,10]. It has been reported that spirulina aqueous extract plays critical roles in the process of reducing oxidative damage [11,12], as well as a wound healing potential on normal human primary dermal fibroblasts [13]. In addition, spirulina can also promote the proliferation of stem cells [14]. Combined with the skin cream, spirulina crude extract was demonstrated to promote the wound healing of human keratinocyte cells (HS2) and human fibroblast cells (L929) [15]. Moreover, C-phycocyanin, purified from spirulina, has been reported to promote mouse wound healing and fibroblasts migration [16]. Our previous study has reported that SPCP contains C-phycocyanin α chain. Meanwhile, our results demonstrate that SPCP promoted the viability of CCD-986sk cells. The epidermal growth factor receptor (EGFR)/mitogen-activated protein kinase (MAPK) signaling pathway was activated in this process [17]. As is known, the activated EGFR can recruit PI3K, then phosphorylate and activate Akt [18]. PI3K/Akt signaling pathway has a crucial role in cell proliferation [19,20,21]. In addition, cell cycle and cell growth can be regulated by the downstream factors of PI3K/Akt signaling pathway, which are activated by phosphorylated Akt [22]. Recent studies suggest that PI3K/Akt signaling pathway participates in the proliferation of fibroblast [23,24]. In addition, PI3K/Akt signaling pathway was activated in the process of ozone oil promoting the mouse skin wound healing. *Calendula officinalis* tincture stimulated the proliferation of fibroblasts depended on PI3K/Akt signaling pathway [25]. However, to the best of our knowledge, whether the PI3K/Akt signaling pathway is involved in the effect of SPCP on the proliferation and migration of CCD-986sk cells is unknown.

Herein, the purpose of this study was to investigate the effect of SPCP on human dermal fibroblasts proliferation and migration, and further reveal its molecular mechanisms. The main findings suggested that SPCP can promote the proliferation and migration of CCD-986sk cells, and that the PI3K/Akt signaling pathway plays a positive and important role in these processes.

## 2. Results

### 2.1. Effect of SPCP on Proliferation of CCD-986sk Cells

**Figure 1:**
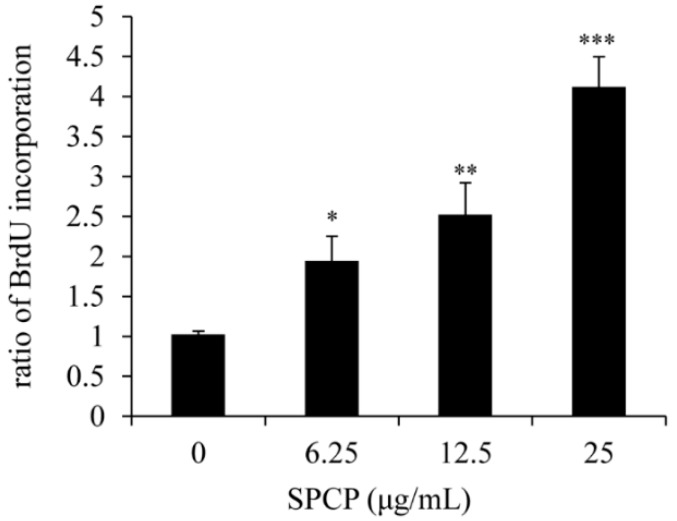
The treatment of spirulina crude protein (SPCP) enhanced the proliferation of CCD-986sk cells. CCD-986sk cells were incubated with various concentrations of SPCP for 24 h and then the cell proliferation was determined by BrdU assay. The results are presented as the mean ± standard deviation of three independent experiments. * *p* <0.05, ** *p* < 0.01, *** *p* <0.001 compared to the control group.

To determine the effect of SPCP on the proliferation of CCD-986sk cells, we performed the BrdU assay as shown in Figure 1. We can observe that after being treated with 6.25, 12.5, or 25 μg/mL SPCP, the ratio of BrdU incorporation in CCD-986sk cells was significantly increased by 0.9 ± 0.31 (*p* < 0.05), 1.5 ± 0.4 (*p* < 0.01), and 3.1 ± 0.38 (*p* < 0.001) with respect to the control group, respectively. Thus, we can conclude that the proliferation of CCD-986sk cells can be prompted by the usage of SPCP in a dose-dependent manner.

### 2.2. Effect of SPCP on Migration of CCD-986sk Cells

**Figure 2:**
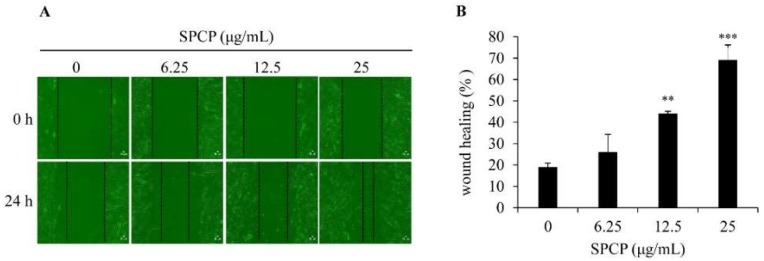
Treatment of SPCP enhanced repair of the scratched area. (**A**) A scratch wound was created using 200 μL pipette tip in a confluent dermal fibroblast. The images were taken at 0 h and 24 h with the indicated concentration of SPCP. The dotted lines show the area where the scratch wound was created. (**B**) A bar graph showing the migration of cells after 24 h following the scratch wound in cells treated with SPCP. The results are presented as the mean ± standard deviation of three independent experiments. ** *p* < 0.01, *** *p* < 0.001 compared to the control group.

To determine the effect of SPCP on the migration of CCD-986sk cells, we performed the wound healing assay. Figure 2A shows the images of wound healing assay on CCD-986sk cells at 0 and 24 h postinjury time with the treatment of SFM or different concentrations of SPCP (6.25, 12.5, and 25 μg/mL). We found that SPCP significantly increased the migration of CCD-986sk cells compared with the control group (Figure 2B, *p* < 0.01 and *p* < 0.001). This result indicated that the treatment of SPCP enhanced the migration and wound closure of CCD-986sk cells in a dose-dependent manner.

### 2.3. Effect of SPCP on the Cell Cycle of CCD-986sk Cells

**Figure 3:**
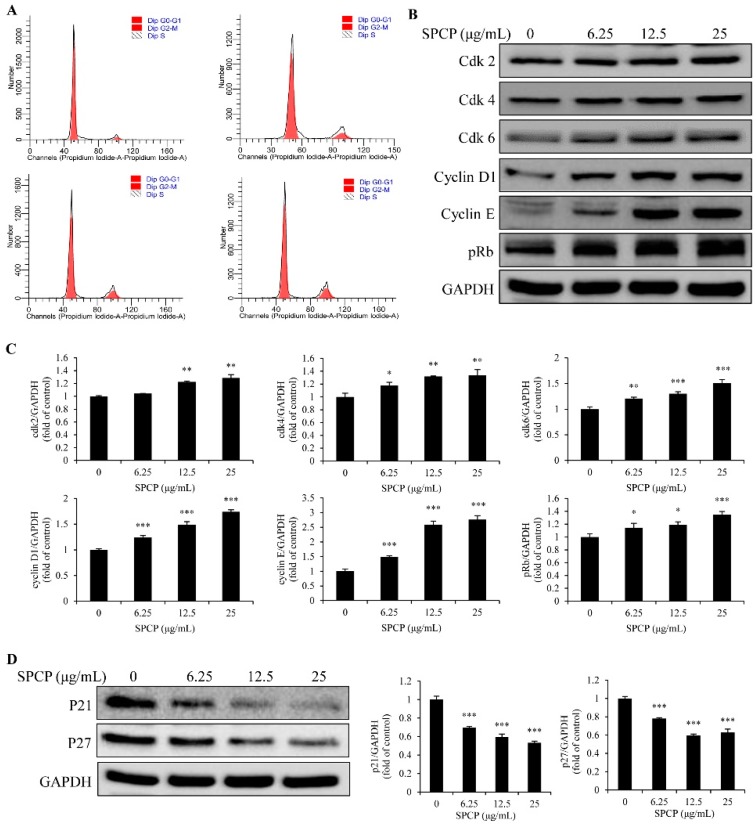
Treatment of SPCP-promoted CCD-986sk cell cycle progression. (**A**) The cell cycle of CCD-986sk was analyzed by flow cytometry. (**B**,**C**) The expression of Cdk2, Cdk4, Cdk6, cyclin D1, cyclin E, and pRb in CCD-986sk cells were measured by western blotting. (**D**) The expression of p21 and p27 in CCD-986sk cells were measured by Western blotting. The results are presented as the mean ± standard deviation of three independent experiments. * *p* < 0.05, ** *p* < 0.01, *** *p* < 0.001 compared to the control group.

The cell cycle of CCD-986sk cells was analyzed by flow cytometry. As shown in Figure 3A and Table 1, after being treated with the different concentrations of SPCP, the accumulation of cells in the G_0_/G_1_ phase was significantly lower than that of control group (*p* < 0.01). However, the percentage of cells in S and G_2_/M phases significantly increased with the treatment of SPCP (*p* < 0.05, *p* < 0.01, and *p* < 0.001). These results indicated that CCD-986sk cells were driven from G_0_/G_1_ to G_2_/M phase by SPCP treatment. Therefore, it can be speculated that the proliferation of CCD-986sk cells was promoted by SPCP.

To confirm the result of cell cycle assay, western blot analysis was performed to detect the expression level of Cdk2, Cdk4, Cdk6, cyclin D1, cyclin E, retinoblastoma protein (pRb), p21, and p27. The results showed that SPCP upregulated the expression of Cdk2, Cdk4, Cdk6, cyclin D1, cyclin E, and pRb (Figure 3B,C, *p* < 0.05, *p* < 0.01, and *p* < 0.001), which are necessary for cell cycle. While the expression of p21 and p27 decreased with the treatment of SPCP (Figure 3D, *p* < 0.001), which are inhibitors of cell cycle. These results indicate that SPCP regulates the expression of cell cycle proteins and promotes the cell cycle progression of CCD-986sk cells.

### 2.4. Treatment of SPCP Activated PI3K/AKT Signaling Pathway in the CCD-986sk Cells

**Figure 4:**
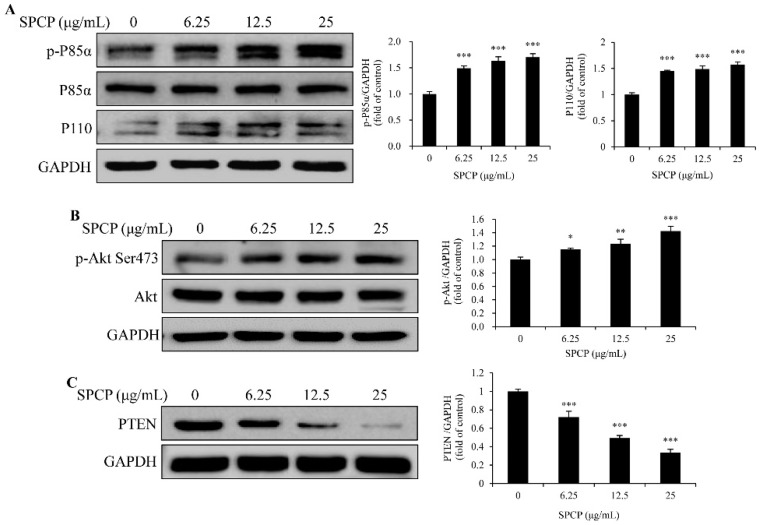
Treatment of SPCP-activated PI3K-Akt signaling pathway in CCD-986sk cells. (**A**) The phosphorylation level of p85α and expression level of p110 in CCD-986sk cells were measured by Western blotting. (**B**) The phosphorylation level of Akt (Ser 473) in CCD-986sk cells were measured by Western blotting. (**C**) The expression level of PTEN in CCD-986sk cells were measured by western blotting. The results are presented as the mean ± standard deviation of three independent experiments. * *p* < 0.05, ** *p* < 0.01, *** *p* < 0.001 compared to the control group.

To determine whether SPCP regulated cell cycle progression and promoted cell proliferation via the PI3K/AKT signaling pathway, we first performed a Western blot analysis. As is known, PI3K consists of a regulatory subunit (p85) and a catalytic subunit (p110) [26,27]. The results showed that the level of phospho-p85α [28] and the expression of p110 in CCD-986sk cells were higher in SPCP-treated groups than the control group (Figure 4A, *p* < 0.001). Meanwhile, the levels of phospho-Akt increased with the treatment of SPCP in CCD-986sk cells (Figure 4B, *p* < 0.05, *p* < 0.01, and *p* < 0.001). PTEN is a natural inhibitor of PI3K-Akt signaling pathway and, after incubation with SPCP, the level of PTEN was decreased in the CCD-986sk cells (Figure 4C, *p* < 0.001). These results demonstrated that SPCP activated the PI3K/Akt signaling pathway and inhibited the expression of PTEN in the proliferation of CCD-986sk cells.

### 2.5. Treatment of SPCP-Activated Mammalian Target of Rapamycin (mTOR) Signaling Pathway in the CCD-986sk Cells

**Figure 5:**
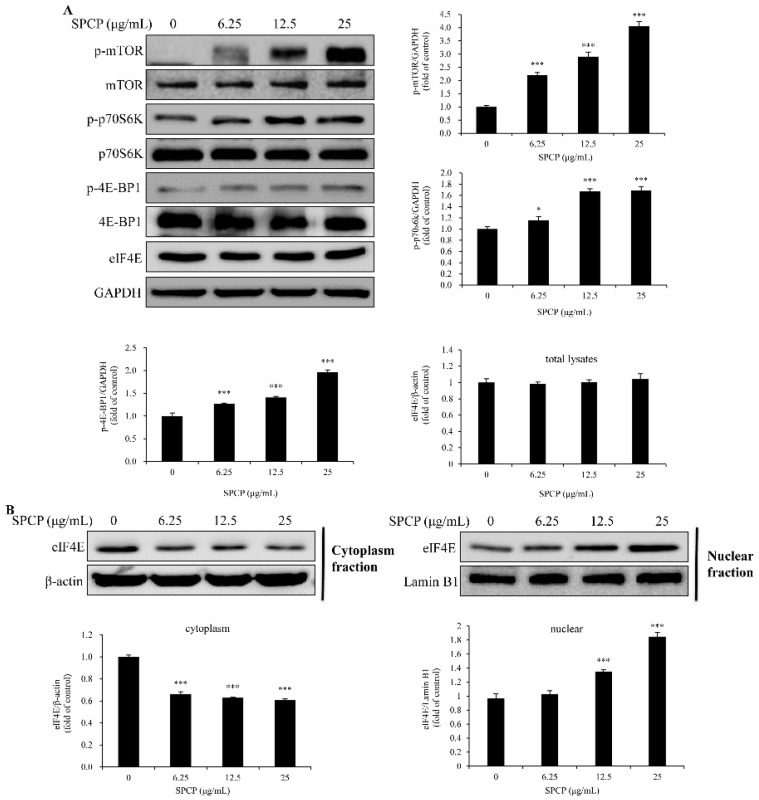
Treatment of SPCP-activated mTOR signaling pathway in CCD-986sk cells. (**A**) The phosphorylation levels of mTOR, p70S6K and 4EBP1 in CCD-986sk cells were measured by Western blotting. (**B**) The cytoplasm and nuclear eIF4E levels in CCD-986sk cells were measured by Western blotting. The results are presented as the mean ± standard deviation of three independent experiments. * *p* < 0.05, *** *p* < 0.001 compared to the control group.

To evaluate whether mTOR was activated by Akt, the phosphorylation level of mTOR was determined by Western blotting. The results showed that the level of phospho-mTOR in CCD-986sk cells was higher in SPCP-treated groups than the control group (Figure 5A, *p* < 0.001). Then, downstream signals were determined. As shown in Figure 5A, the levels of phospho-Eukaryotic translation initiation factor 4E (eIF4E)-binding protein 1 (4E-BP1) and phospho-p70S6k were increased with the treatment of SPCP in CCD-986sk cells (*p* < 0.05, *p* < 0.01).

The complex between 4E-BP1 and the translation factor eIF4E will be disrupted with the phosphorylation of 4E-BP1 [29]. Thus, we examined the expression level of eIF4E. Western blot analysis showed that the expression level of eIF4E was not affected by SPCP in CCD-986sk cells (Figure 5A). However, the concentration of eIF4E was reduced in cytoplasmic (Figure 5B, *p* < 0.001). Moreover, the nuclear eIF4E concentration was significantly increased with the treatment of SPCP in CCD-986sk cells (Figure 5B, *p* < 0.001). These results indicated that the phosphorylation of Akt induced mTOR activation and led to the phosphorylation of p70S6k and 4E-BP1. Further, the phosphorylation of 4EBP1 led to the release of eIF4E and transfer to the nucleus.

### 2.6. Treatment of SPCP Increased the Phosphorylation of Glycogen Synthase Kinase 3 Beta (GSK3β) in the CCD-986sk Cells

**Figure 6:**
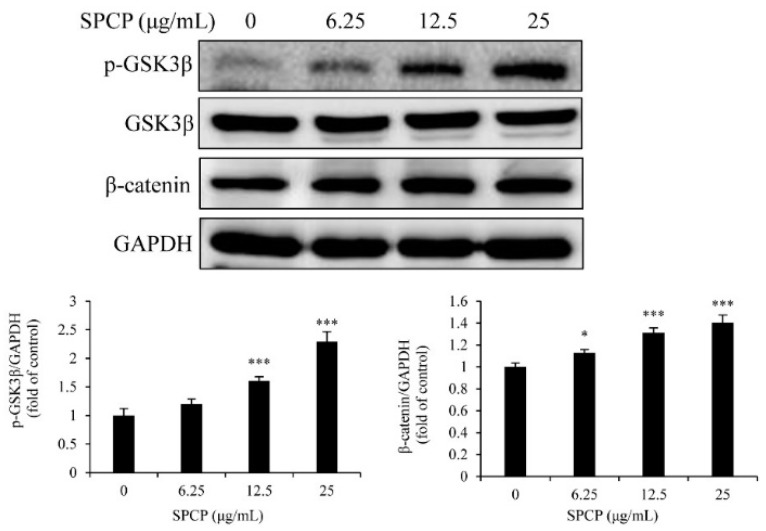
Treatment of SPCP increased the phosphorylation of GSK3β in CCD-986sk cells. The phosphorylation level of GSK3β and expression level of β-catenin in CCD-986sk cells were measured by Western blotting. The results are presented as the mean ± standard deviation of three independent experiments. * *p* < 0.05, *** *p* < 0.001 compared to the control group.

To evaluate whether GSK3β was regulated by Akt, the phosphorylation level of GSK3β was determined by Western blotting. The results showed that SPCP significantly increased the phosphorylation level of GSK3β (Figure 6, *p* < 0.001). Meanwhile, the level of β-catenin increased by the treatment of SPCP (Figure 6, *p* < 0.05, *p* < 0.001). These results indicated GSK3β was inactivated by the activation of Akt and led to the increase of β-catenin.

### 2.7. Inhibition of PI3K Reduced SPCP-Induced Proliferation and Migration of CCD-986sk Cells

**Figure 7:**
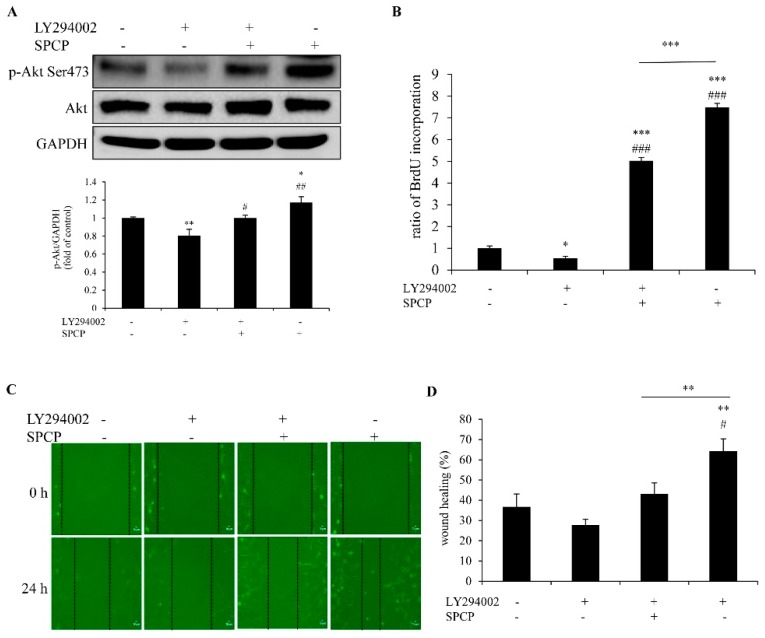
PI3K inhibitor LY294002 inhibited the level of phospho-Akt, proliferation and migration of CCD-986sk cells in SPCP-treated group. CCD-986sk cells were pretreated with PI3K inhibitor LY294002 (50 μmol/L) and then treated with SPCP for 24 h. (**A**) The level of phospho-Akt (Ser 473) was analyzed by Western blotting. (**B**) The proliferation of CCD-986sk cells was measured by BrdU assay. (**C**) Cell migration of CCD-986sk cells was measured by wound healing assay. (**D**) A bar graph showed the migration of cells after 24 h following the scratch wound in cells treated with SPCP. The results are presented as the mean ± standard deviation of three independent experiments. * *p* < 0.05, ** *p* < 0.01, *** *p* < 0.001 compared with control group. ^#^ *p* < 0.05, ^##^ *p* < 0.01, ^###^ *p* < 0.001 compared with inhibitor group.

To further determine whether SPCP promoted CCD-986sk cell proliferation and migration depends on PI3K; the PI3K inhibitor LY294002 (50 μmol/L) was used to pretreat cells for 1 h. The level of phosphorylated Akt was detected by western blot analysis. We found that it was significantly increased by the treatment of SPCP and decreased by the pretreatment of LY294002 in CCD-986sk cells (Figure 7A, *p* < 0.05, *p* < 0.01).

Cell proliferation was measured by BrdU assay. The proliferation of CCD-986sk cells was significantly increased with the addition of SPCP (25 μg/mL) compared with control group (Figure 7B, *p* < 0.001). However, this increase was blocked by pretreatment of LY294002 (Figure 7B, *p* < 0.001). This result showed that LY294002 blocked the cell proliferation which was induced by SPCP.

The wound healing assay showed that the migration of CCD-986sk cells decreased significantly after pretreatment with LY294002 in the SPCP-treated group (Figure 7C,D, *p* < 0.01). This result indicated that the cell migration induced by SPCP was blocked by LY294002.

Taken together, these results further demonstrated that PI3K-Akt signaling pathway was involved in SPCP-promoted CCD-986sk cells proliferation and migration.

## 3. Discussion

In general, skin is regarded as a barrier to protect the inner organs from environmental damage, which makes it extremely vulnerable to different types of lesions [30]. Skin wounds can cause the inner organ to become ill with poor nutrition and even death [31]. Thus, there is an urgent need to find an effective method to promote skin wound healing. As is known, there are many complex processes in wound healing, such as new tissue formation and remodeling. It has been shown that the proliferation and migration of fibroblasts is necessary for new tissue formation and remodeling [32]. Therefore, dermal fibroblasts play a vital role in cutaneous wound healing.

It is reported that spirulina extract has potential for promoting wound healing. Jung et al. [33] found that spirulina water extract had a positive effect on rat fibroblasts viability and proliferation. Bari et al. [34] showed that *Arthrospira platensis* (spirulina) aqueous extract which, combined with silk sericin, had the ability to induce a wound closure of a human fibroblast. Syarina et al. [13] demonstrated that spirulina aqueous extract stimulated the proliferation of human dermal fibroblast. In this study, we observed that SPCP significantly promoted the proliferation and migration of CCD-986sk cells. In addition, the proliferation of dermal fibroblasts is one of the most important factors for skin wound healing. An important factor to control cell proliferation is the interface between the cell cycle signaling system and growth factor signaling pathways [35,36]. Hence, the cell cycle was analyzed by flow cytometry. Our results showed that the percentage of cells in G_0_/G_1_ was decreased in SPCP-treated groups compared with the control group. Meanwhile, the number of cells in S and G_2_/M phases was increased in SPCP-treated groups compared with the control group. These results demonstrated that SPCP stimulated cells to enter S and G_2_/M phases from the G_0_/G_1_ phase, which is an indication of the proliferation of CCD-986sk cells [37]. As is known, the cell cycle is controlled by a number of signaling systems [38]. The expression of cell cycle related proteins, such as cyclin D, cyclin E, Cdk2, Cdk4, Cdk6, pRb and the inhibitors of cell cycle p21 and p27 were detected by western blotting. Our study found that the expression of cyclin D, cyclin E, Cdk2, Cdk4, Cdk6 and pRb were increased with the treatment of SPCP. The expression of p21 and p27, which are inhibitors of the cell cycle [39], was decreased by the treatment of SPCP. The cell cycle is driven by proliferation signaling pathways and the cyclins are the primary targets. Cyclin D combines with Cdk4 or Cdk6 to form an active complex, and cyclin E combines with Cdk2 to form an active complex. These two complexes control G_1_ progression and DNA synthesis to regulate the cell cycle [40]. Our results indicate that SPCP promotes the cell cycle of CCD-986sk cells by promoting the G_0_/G_1_ phase to enter S and G_2_/M phases and activating cell cycle-related proteins.

PI3K/Akt signaling pathway is one of the important pathways that regulates cell cycle and promotes cell proliferation, which can be recruited by activated EGFR and then activated the downstream signaling [31,32,33,34,35,36,37,38,39,40,41,42,43]. Further, enhanced phosphorylation of the PI3K/Akt signaling pathway can promote the proliferation of cells [44,45]. Our recent study showed that SPCP activated EGFR signaling in CCD-986sk cells [17]. Thus, in this study, we investigated whether the PI3K/Akt signaling pathway involved in SPCP-treated CCD-986sk cells. We found that the levels of phospho-p85α, p110, and phospho-Akt in CCD-986sk cells were enhanced by SPCP treatment. In addition, the expression level of PTEN, which is one of the natural inhibitors of PI3K/Akt signaling pathway was inhibited by SPCP treatment. It has been reported that the phosphorylation of Akt induces the activation of mTOR and reduces the activation of GSK3β [46,47]. mTOR signaling has an important role in regulating cell proliferation, and it has two important phosphorylation substrates: 4EBP1 and p70S6K [48]. As is known, one of the crucial factors for controlling cell proliferation is the regulation of translation. eIF4E plays a key role in the regulation of translation, while translation is inhibited by the binding of 4EBP1 with eIF4E. However, when 4EBP1 is phosphorylated by activated mTOR, eIF4E is released and promotes protein synthesis [49]. In addition, p70S6K is another one phosphorylation substrate of mTOR, which controls translation [50]. In this study, we found that the phosphorylation levels of 4EBP1 and p70S6K were enhanced by SPCP. Meanwhile, SPCP increased the eIF4E of nucleus in CCD-986sk cells. Otherwise, β-catenin has been reported to affect the activity of various transcription factors, which can be degraded by GSK3β [51]. However, Akt can reduce the activity of GSK3β by phosphorylation of Ser-9 [46]. Thus, inhibition of GSK3β leads to the increase of β-catenin. Further, β-catenin associates with transcription factors and initiates transcription, and thereby regulates the expression of various genes including those proteins involved in cell cycle [52]. This study reported that SPCP increased the phosphorylation of GSK3β. Meanwhile, the expression level of β-catenin was enhanced by SPCP. These results demonstrated that PI3K/Akt/mTOR signaling pathway plays an important role in the migration and proliferation of CCD-986sk cells which induced by SPCP. To further investigate the role of Akt in SPCP-induced CCD-986sk cell migration and proliferation, the inhibitor LY294002 was used to pretreat cells. The level of phospho-Akt was decreased with the pretreatment of LY294002 compared with SPCP-treated cells. The BrdU assay result showed that LY294002 significantly inhibited SPCP-induced CCD-986sk cell proliferation. Meanwhile, the migration of SPCP-treated CCD-986sk cells was blocked by LY294002. Therefore, the proliferation and migration of CCD-986sk cells which were promoted by SPCP via the phosphorylation and activation of Akt.

In conclusion, SPCP can promote the human dermal fibroblasts proliferation and migration, which are important factors of wound healing. The PI3K/Akt signaling pathway is involved in this process. The results of this study provide important evidence and reveal the mechanism of the wound healing potential of SPCP. Furthermore, the development of a human fibroblast migration and proliferation activated by SPCP and the finding that activation of the PI3K/Akt signaling pathway provide a potential application of SPCP in skin wound healing.

## 4. Materials and Methods

### 4.1. Preparation of Spirulina Crude Protein

Spirulina (New Zealand Nutritionals (2004) Ltd., Burnside Christchurch, New Zealand) was churned up in distilled water for 4 h at 40 g/L. The mixture was centrifugated (2400× g, 4 °C, 10 min) and then mixed with triple of the volume of ethanol. After incubation at 4 °C for 4 h, the solution was centrifugated (2400× g, 4 °C, 10 min) and subsequently filtered. Next, the solution was concentrated and precipitated overnight with (NH_4_)_2_SO_4_ (80% saturation) at 4 °C. The precipitate was dissolved in distilled water and dialyzed using rotary evaporator. The dialysate (1000 μg/mL) was freeze-dried and stored in −70 °C.

### 4.2. Cell Culture

Human dermal fibroblasts CCD-986sk cells (ATCC CRL-1947; American Type Culture Collection, Manassas, VA, USA) derived from normal female skin tissue were grown in Dulbecco’s Modified Eagle’s Medium (DMEM) with 10% fetal bovine serum and 1% penicillin/streptomycin. Cells were grown and maintained at 37 °C and 5% CO_2_ in a humidified cell incubator. The medium was replaced every other day and passaged when the cells attained ~60–80% confluence.

### 4.3. BrdU Assay

Cell proliferation was determined using a Cell Proliferation ELISA, BrdU (colorimetric) kit (Sigma-Aldrich; Merck KGaA, Darmstadt, Germany). CCD-986sk cells were seeded into a 96-well plate at a density of 0.5 × 10^4^ cells/well. After 24 h of incubation, cells were incubated with serum-free (SFM) medium for 4 h at 37 °C and then treated with different concentrations of SPCP (0, 6.25, 12.5, and 25 μg/mL in SFM) for 24 h. For inhibitor detection, CCD-986sk cells were pretreated with LY294002 (50 μmol/L) for 1 h. Subsequently, 10 μL of BrdU labeling solution were added to each well and incubated for additional 2 h at 37 °C. The BrdU labeling medium was removed, and 200 μL/well of FixDenat was added. After 30 min of incubation at room temperature, the FixDenat solution was removed and 100 μL of anti-BrdU-POD working solution was added to each well. After incubation for 90 min at room temperature, each well was thrice-washed with 200 μL of washing solution (PBS, 1×). Each well was then incubated with 100 μL of Substrate solution for 5–30 min at room temperature until the color is sufficient for photometric detection. Absorbance at 370 and 492 nm were measured using a Synergy HTX microplate reader (BioTek Instruments, Inc., Winooski, VT, USA). Data are expressed as a ratio of absorbance (A_370 nm_ − A_492 nm_) in treated cells compared with the SFM-treated control.

### 4.4. Wound Healing Assay

CCD-986sk cells were seeded in six well plates and grown under standard culture conditions. The culture medium was replaced every other day as described above. After confluence, the cells were incubated with serum free medium (SFM) for 4 h. A single wound was then created with a sterile 200 μL plastic pipette tip in the center of the well. The cells were washed with PBS twice to remove the cellular debris and treated by different concentrations of SPCP (0, 6.25, 12.5, and 25 μg/mL) for 24 h. The wound was captured by an inverted microscope equipped with a digital camera at 0 and 24 h, respectively. The size of the wound healing was measured using Image J software (version 1.40; National Institutes of Health, Bethesda, MD, USA). It was expressed as a percentage of the initial distance of the wound.

### 4.5. Flow Cytometry

Cell cycle progress was determined by flow cytometry using BD Cycletest^TM^ Plus DNA Reagent Kit (becton, dikinson and company). Cells were starved in SFM for 4 h and then treated with different concentrations of SPCP (0, 6.25, 12.5, and 25 μg/mL) for 24 h. Cells were harvested and washed by buffer solution thrice. Counting the cells and adjusting the concentration to 1.0 × 10^6^ cells/mL with buffer solution were performed out. After centrifugation, the cells were incubated with solution A (trypsin buffer) at room temperature (20–25 °C) for 10 min and then incubated with solution B (trypsin inhibitor and RNase buffer) at room temperature (20–25 °C) for another 10 min. Ultimately, the cells were incubated with solution C (PI stain solution) for 10 min in the dark on ice. Cell cycle was detected by the BD FACSVerse^TM^ system (Becton, Dickinson and Company, USA) and analyzed using ModFit (version 3.1; Verity Software House, Topsham, ME, USA).

### 4.6. Nuclear and Cytoplasmic Lysates

CCD-986sk cells were treated as described above. Nuclear and cytoplasmic extractions were separated using the NE-PER Nuclear and Cytoplasmic Extraction Reagents (Pierce Biotechnology, Inc., Rockford, IL, USA) according to the manufacturer’s instructions. The protein concentration was analyzed using a Bicinchoninic acid protein assay kit (Pierce; Thermo Fisher Scientific, Inc.). Equal amount of proteins was denatured at 100 °C for 5 min with sodium dodecyl sulfate (SDS) sample buffer containing dithiothreitol (DTT).

### 4.7. Whole Cell Lysates

CCD-986sk cells were treated as described above. For inhibitor detection, CCD-986sk cells were pretreated with LY294002 (50 μmol/L) for 1 h. After treatment, cells were lysated by radioimmunoprecipitation lysis buffer (iNtRON Biotechnology) with 1% protease inhibitor at 4 °C for 30 min. Cells were collected by scraping and proteins were isolated by centrifugation (18,341× g, 4 °C, 10 min). The protein concentration was analyzed using Bicinchoninic acid protein assay kit (Pierce; Thermo Fisher Scientific, Inc.). Equal amount of proteins was denatured at 100 °C for 5 min with sodium dodecyl sulfate (SDS) sample buffer containing dithiothreitol (DTT).

### 4.8. Western Blotting

Proteins were separated by SDS-PAGE and then transferred to PVDF membranes (Millipore, Milford, CT, USA). The membranes were washed with methyl alcohol, and then blocked with 1% bovine serum albumin (BSA) in TBS-T (10 mM Tris-HCl, 150 mM NaCl (pH 7.5), and 0.1% Tween-20) for 2 h. Membranes were incubated with primary antibody overnight at 4 °C. After washing two times, the membranes were incubated with secondary antibody for 2 h at room temperature. The second antibodies were horseradish peroxidase (HRP)-conjugated anti-rabbit IgG (Cell Signaling Technology, Inc., Beverly, MA, USA, cat. no. 7074S), donkey anti-goat IgG (Bethyl Laboratories, Inc., Beverly, MA, USA, cat. no. A50-101p), and anti-mouse IgG (Cell Signaling Technology, Inc., cat. no. 7076S). The following primary antibodies obtained from Santa Cruz Biotechnology, Inc. were used: Rabbit anti-Cdk2 antibody (cat. no. sc-163), rabbit anti-Cdk4 antibody (cat. no. sc-601), rabbit anti-Cdk6 antibody (cat. no. sc-177), mouse anti-cyclin D1 antibody (cat. no. sc-8396), rabbit anti-cyclin E antibody (cat. no. sc-481), mouse anti-p-Rb antibody (cat. no. sc-377528), mouse anti-p21 antibody (cat. no. sc-271532), rabbit anti-p27 antibody (cat. no. sc-528), goat anti-phosphorylated (p)-PI3-kinase p85α antibody (cat. no. sc-12929), mouse anti-PI3-kinase p85α antibody (cat. no. sc-1637), mouse anti-PI3-kinase p110 antibody (cat. no. sc-8010), mouse anti-p-Akt1/2/3 antibody (cat. no. sc-514032), rabbit anti-Akt1/2/3 antibody (cat. no. sc-8312), mouse anti-p-mammalian target of rapamycin (mTOR) antibody (cat. no. sc-293132), rabbit anti-mTOR antibody (cat. no. sc-8319), mouse anti-p-p70 S6 kinase α (p70S6k) antibody (cat. no. sc-8416), mouse anti-p70 S6 kinase α antibody (cat. no. sc-8418), mouse antiphosphatase, tensin homolog (PTEN) antibody (cat. no. sc-8416), mouse anti-p-4EBP1 antibody (cat. no. sc-293124), mouse anti-4EBP1 antibody (cat. no. sc-9977), mouse anti-eIF4E antibody (cat. no. sc-514875), mouse anti-p-GSK-3β antibody (cat. no. sc-373800), mouse anti-GSK-3β antibody (cat. no. sc-377213), goat anti-β-catenin antibody (cat. no. sc-1496), mouse anti-β-Actin (cat. no. sc-47778), mouse anti-Lamin B1 antibody (cat. no. sc-377000), and rabbit anti-GAPDH antibody (cat. no. sc-25778). An enhanced chemiluminescence Western blot kit (Thermo Fisher Scientific, Rockford, IL, USA) was used for color development. The protein bands were detected using bioanalytical imaging system (Azure Biosystems, Dublin, CA, USA). The density of these bands which normalized to GAPDH, β-Actin or Lamin B1 was analyzed using Multi-Gauge software, version 3.0 (Fujifilm Life Science, Tokyo, Japan).

### 4.9. Statistical Analysis

Every assay is performed at least three independent experiments. Data, which is calculated using Microsoft Excel, is expressed as mean ± standard deviation. The differences among multiple groups were evaluated using one-way analysis of variance followed by Bonferroni post-hoc test using SPSS statistical software for Windows, version 20.0 (IBM Corp., Armonk, NY, USA). The values of *p* < 0.05 were considered as statistical significance.

## Figures and Tables

**Table 1:** Effects of SPCP on the cell cycle of CCD-986sk cells.

SPCP (μg/mL)	G_0_/G_1_ (%)	S (%)	G_2_/M (%)	S + G_2_/M (%)
0	94.01 ± 2.494	1.84 ± 0.875	4.15 ± 1.759	5.99 ± 2.493
6.25	87.47 ± 3.604	3.513 ± 0.587 *	9.087 ± 3.500	12.6 ± 3.586 *
12.5	83.0233 ± 3.647 **	3.647 ± 0.387 **	13.33 ± 1.970 **	16.977 ± 2.350 **
25	81.113 ± 2.045 **	3.73 ± 0.732 **	15.157 ± 1.339 **	18.887 ± 2.045 ***

The results are presented as the mean ± standard deviation of three independent experiments. * *p* < 0.05, ** *p* < 0.01, *** *p* < 0.001 compared to the control group.
